# A comparison of five Illumina, Ion Torrent, and nanopore sequencing technology-based approaches for whole genome sequencing of SARS-CoV-2

**DOI:** 10.1007/s10096-023-04590-0

**Published:** 2023-04-05

**Authors:** Ellen C. Carbo, Kees Mourik, Stefan A. Boers, Bas Oude Munnink, David Nieuwenhuijse, Marcel Jonges, Matthijs R. A. Welkers, Sebastien Matamoros, Joost van Harinxma thoe Slooten, Margriet E. M. Kraakman, Evita Karelioti, David van der Meer, Karin Ellen Veldkamp, Aloys C. M. Kroes, Igor Sidorov, Jutte J. C. de Vries

**Affiliations:** 1 Clinical Microbiological Laboratory, Department of Medical Microbiology, Leiden University Medical Center, Leiden, The Netherlands; 2 Department of Viroscience, Erasmus Medical Centre, Rotterdam, The Netherlands; 3 Department of Medical Microbiology and Infection Prevention, Amsterdam University Medical Centers, University of Amsterdam, Amsterdam, The Netherlands; 4 GenomeScan B.V, Leiden, The Netherlands

**Keywords:** Whole genome sequencing, SARS-CoV-2, Benchmark

## Abstract

Rapid identification of the rise and spread of severe acute respiratory syndrome coronavirus 2 (SARS-CoV-2) variants of concern remains critical for monitoring of the efficacy of diagnostics, therapeutics, vaccines, and control strategies. A wide range of SARS-CoV-2 next-generation sequencing (NGS) methods have been developed over the last years, but cross-sequence technology benchmarking studies have been scarce. In the current study, 26 clinical samples were sequenced using five protocols: AmpliSeq SARS-CoV-2 (Illumina), EasySeq RC-PCR SARS-CoV-2 (Illumina/NimaGen), Ion AmpliSeq SARS-CoV-2 (Thermo Fisher), custom primer sets (Oxford Nanopore Technologies (ONT)), and capture probe-based viral metagenomics (Roche/Illumina). Studied parameters included genome coverage, depth of coverage, amplicon distribution, and variant calling. The median SARS-CoV-2 genome coverage of samples with cycle threshold (Ct) values of 30 and lower ranged from 81.6 to 99.8% for, respectively, the ONT protocol and Illumina AmpliSeq protocol. Correlation of coverage with PCR Ct values varied per protocol. Amplicon distribution signatures differed across the methods, with peak differences of up to 4 log_10_ at disbalanced positions in samples with high viral loads (Ct values ≤ 23). Phylogenetic analyses of consensus sequences showed clustering independent of the workflow used. The proportion of SARS-CoV-2 reads in relation to background sequences, as a (cost-)efficiency metric, was the highest for the EasySeq protocol. The hands-on time was the lowest when using EasySeq and ONT protocols, with the latter additionally having the shortest sequence runtime. In conclusion, the studied protocols differed on a variety of the studied metrics. This study provides data that assist laboratories when selecting protocols for their specific setting.

**Supplementary information:**

The online version contains supplementary material available at 10.1007/s10096-023-04590-0.

## Introduction

Genomic surveillance of severe acute respiratory syndrome coronavirus 2 (SARS-CoV-2) has proven critical for early detection of the rise and spread of SARS-CoV-2 variants of concern, for monitoring and developing effective diagnostic, therapeutic, and preventive strategies [1]–[3]. In addition, genomic surveillance assists in contact tracing, transmission tracking at population level, and public-health decision-making [4]. The widespread application of genomics for pandemic surveillance is exemplified by more than 15 million SARS-CoV-2 sequences deposited in the GISAID repository as of February 2023 [5].

A wide range of SARS-CoV-2 next-generation sequencing (NGS) technologies and protocols have been developed and adapted since the first genome sequence was generated using a metagenomic approach [6]–[8]. SARS-CoV-2 whole genome sequencing (WGS) protocols have been improved to increase the technical performance, including sensitivity and genome coverage, and logistical aspects have also been addressed, such as scalability and hands-on time [9]–[12]. Studies have been published on SARS-CoV-2 WGS with innovative protocol adaptations in order to decrease the error rate and the turnaround time by combining PCR and tagging steps [12] [13]. However, these studies have been typically focused on the technology developed by the authors, whereas comparison of a novel protocol with other methods is limited. Benchmark studies of SARS-CoV-2 genome sequencing technologies are limited and generally restricted to comparison of protocols for the single type of sequencing technology available at the study site of the authors [14]–[18] or cross-platform studies limited to only amplicon sequencing protocols [19]–[21]. In contrast, cross-platform studies including short and long read sequencing platforms and metagenomics remain relatively scarce and limited to a maximum of two different sequence platforms [22]. A recent external quality assessment (EQA) report assessed the outcome of complete workflows from nucleic acid extraction to the reported consensus sequence by testing SARS-CoV-2 cultured isolates; however, no detailed distinction between the different workflow components could be made [20].

**Fig. 1:**
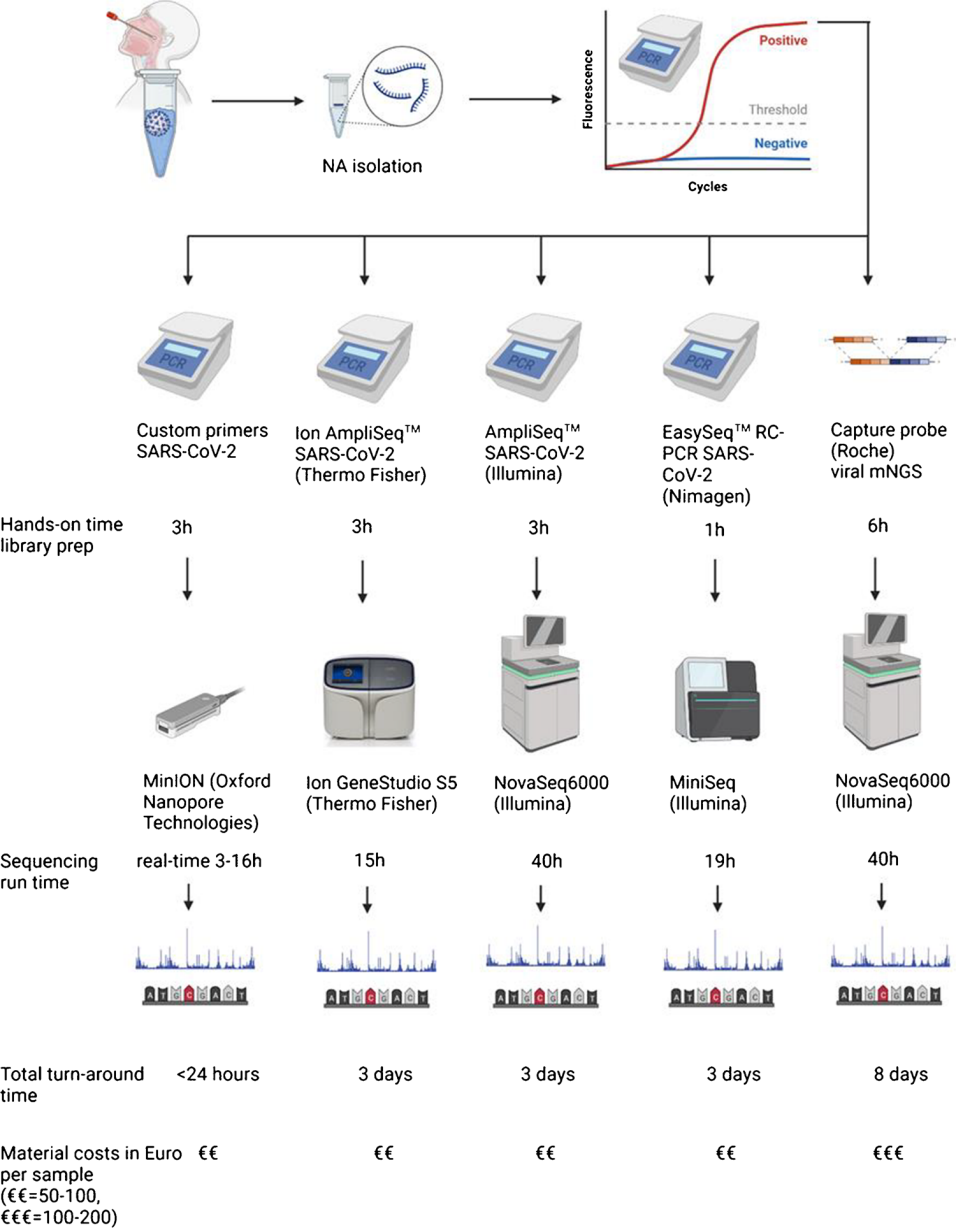
Schematic overview of the design, workflow, and technologies adopted in this study with their hands-on and sequencing turnaround time. Twenty-six respiratory samples, mainly nasopharyngeal swabs and tracheal aspirates, were tested by five SARS-CoV-2 WGS protocols. PCR Ct values ranged from 13.9 to 33.6. To exclude potential variability resulting from different nucleic acid extraction methodologies, the extraction method used was identical for all five protocols. Four protocols were tiled amplicon based, one protocol was capture probe based, targeting all viruses known to infect vertebrates designed in 2015 [29] but shown to cover > 99% of the SARS-CoV-2 genome [30] due to similarity with bat coronaviruses and the variability incorporated in the probe design. In order to minimize potential differences resulting from variation in bioinformatic analyses tools and settings, a uniform pipeline for sequence data from Illumina and Ion platforms, for ONT data, platform-specific tools handling higher error rates were used to gain optimal results from this type of dataset (Suppl. Figure 1). Created using Biorender.com

Here, we describe a cross-platform benchmark study that includes Illumina, Ion Torrent, and nanopore-based SARS-CoV-2 sequencing technologies in one study. Five protocols (Fig. 1) employing a diversity of sequencers with a wide range of throughput, accuracy, and runtime were compared using clinical samples. The performance was studied by comparing genome coverage, read depth, amplicon distribution, variant calling, and the proportion of on-target reads.

## Methods

### Sample selection

In total, 26 SARS-CoV-2 PCR-positive samples of 24 patients were selected: nine tracheal aspirates, 16 nasopharyngeal/throat swabs, and one lung lymph node biopsy. Fifteen of these samples were obtained for cluster identification. Samples were retrospectively included to be tested with five WGS protocols. Samples were previously sent to the Clinical Microbiological Laboratory of the Leiden University Medical Center (LUMC, The Netherlands) for nucleotide extraction and SARS-CoV-2 E-gene PCR testing [23] in the period March–October 2020 (Wuhan-like viruses circulating). After nucleotide extraction and PCR, samples were stored at $-80$ ° C until further distribution between different centers for WGS analysis. In total, 26 samples with a wide range of Ct values (13.9–33.6, confirmed by retesting) were included to assess the performance of each of the five WGS protocols. The range and distribution of PCR Ct values were chosen based on relevance for routine clinical practice. Since clinical uncultivated samples were used; the available volume restricted the comparison to five different methodologies, without repeated measurements.

### Ethical approval

Approval was obtained from the ethical committee of the LUMC (B20.002, Biobank Infectious Diseases 2020–03) and the Institutional Review Board of the LUMC for observational COVID-19 studies (CoCo 2021–006).

### Extraction of nucleic acids

To exclude potential variability resulting from different nucleic acid extraction methodologies, the extraction method used was identical for all five protocols. Nucleic acids were extracted from 200 μl input material using the MagNApure96 DNA and Viral NA small volume extraction kit on the MagNA Pure 96 System (Roche Diagnostics, Almere, The Netherlands) with 100 μl output eluate.

### *SARS-CoV-2 sequencing protocols (see also Fig. **1**)*

#### AmpliSeq SARS-CoV-2 sequencing (Illumina)

Libraries were prepared using the AmpliSeq™ SARS-CoV-2 Research Panel for Illumina®, which is a targeted RNA/cDNA amplicon assay for epidemiological research of the SARS-CoV-2 virus. This panel contains a two-pool design of 247 amplicons/primer pairs (pool 1: 125 amplicons, pool 2: 122 amplicons). In total, 237 amplicons were SARS-CoV-2 targets while the remaining amplicons mapped to five different regions of the human genome and were used as control. The amplicons’ lengths ranged from 125 to 275 bp.

From each sample, 15 μl of eluate was concentrated using the SpeedVac vacuum concentrator (Eppendorf, Hamburg, Germany). Samples were then dissolved in 10 μl AmpliSeq cDNA synthesis master mix. Next, the AmpliSeq cDNA Synthesis for Illumina Kit (Illumina) was used to reverse transcribe RNA to cDNA. Amplicon primer pools of the AmpliSeq™ SARS-CoV-2 Research Panel for Illumina® were subsequently added to each sample. cDNA target amplification reaction was performed according to manufacturer’s instructions, followed by partial digestion of primer dimers. AmpliSeq CD indexes were then ligated, and further library PCR amplification was performed. The libraries were purified with the Agencourt™ AMPure™ XP Reagent (Beckman Coulter). The final quality and quantity of each barcoded cDNA library were determined using the Fragment Analyzer (Agilent). From all amplified libraries, 2 μl was pooled and loaded for a short sequencing run to indicate the size of the intact libraries. Based on the indicative read counts, equimolar amounts of each sample were pooled (1.1 nM) and submitted for DNA sequencing using the NovaSeq 6000 system (Illumina, San Diego, CA, USA) according to manufacturer’s protocols. Approximately 10 million 150 bp paired-end reads were obtained per sample. Data processing was performed in real time by the NovaSeq Control Software v1.7.

#### EasySeq RC-PCR SARS-CoV-2 sequencing (NimaGen/Illumina)

Libraries were prepared using the EasySeq RC-PCR SARS-CoV-2 kit version 4.02 (NimaGen) for Illumina as described by Coolen et al. [12]. cDNA synthesis was performed using the iScript™ Advanced cDNA Synthesis Kit (Bio-Rad) according to manufacturer’s instructions using 10 μl of eluate. This version of the EasySeq RC-PCR SARS-CoV-2 kit uses 154 designed primer pairs (pools A and B) with a tiling strategy, resulting in approximately 435 bp size amplicons. The EasySeq protocol enables a one-step procedure for adding SARS-CoV-2 target specific PCR primers, sequence adapters, and unique dual indices (UDIs) by hybridization of the SARS-CoV-2 primers with universal primers that include adapters and UDIs. After the PCR with 5 μl cDNA as input, samples were pooled based on Ct value into pools A and B, which were individually cleaned using AmpliClean™ Magnetic Bead PCR Clean-up Kit (NimaGen, Nijmegen, The Netherlands). Subsequently, quantification was performed using the Qubit double-strand DNA (dsDNA) high-sensitivity assay kit on a Qubit 4.0 instrument (Life Technologies) and pools A and B were combined. Sequencing was performed on Illumina MiniSeq® using a Mid Output Kit (2 × 149 or 2 × 151 cycles) (Illumina, San Diego, CA, USA) by loading 0.8 pM on the flow cell, obtaining approximately 50,000 paired-end reads per sample. The sequence runs were conducted using a balanced library pooling strategy based on estimated cDNA input according to the manufacturer’s protocol.

#### Ion AmpliSeq SARS-CoV-2 sequencing (Thermo Fisher)

The Ion AmpliSeq SARS-CoV-2 research panel supplied by Thermo Fisher Scientific contained 247 primer pairs designed to cover the SARS-CoV-2 genome with 125 to 275 bp overlapping amplicons. For cDNA synthesis, the SuperScript VILO cDNA Synthesis Kit (11,754,050, Thermo Fisher Scientific, The Netherlands) was used according to manufacturer’s instructions using 7 μl of diluted nucleic acid solution to an estimated input of 100 copies/reaction using nuclease free water (AM9939, Ambion, Thermo Fisher Scientific, The Netherlands). SARS-CoV-2 whole genome amplification, adapter ligation, and purification were performed using the Ion AmpliSeq SARS-CoV-2 Insight Research Assay (A51305, Thermo Fisher Scientific, The Netherlands) according to manufacturer’s instruction. Libraries were quantified using the Ion Library TaqMan Quantitation Kit (4,468,802, Thermo Fisher Scientific, The Netherlands) according to manufacturer’s instructions. Samples were then sequenced on an Ion GeneStudio S5 system (Thermo Fisher Scientific, The Netherlands) using an Ion 540 chip (Thermo Fisher Scientific, The Netherlands), obtaining approximately up to 1 million paired-end reads per sample.

#### Custom primers with MinION sequencing (ONT)

A SARS-CoV-2 specific multiplexed PCR for nanopore sequencing was performed using custom-made primers as previously described [4], for maximum flexibility and rapid adaptation of primers for novel variants. In short, primers for 89 overlapping amplicons spanning the whole SARS-CoV-2 genome were designed using primal [24]. The amplicon length was approximately 500 bp with a 75 bp overlap between the different amplicons. cDNA was transcribed using SuperScript III Reverse Transcriptase (Invitrogen, Darmstadt, Germany) [25]. Libraries were generated using the native barcode kits from Oxford Nanopore Technologies (EXP-NBD104, EXP-NBD114, EXP-NBD196, and SQK-LSK109) using 5 μl cDNA as input and sequenced on a R9.4 flow cell multiplexing 96 samples per sequence run [4]. On average, 68 k reads with an average size of 423 bp were obtained per sample.

#### Capture probe (Roche) with viral metagenomic NGS (Illumina)

The viral metagenomic NGS protocol has previously been described [26]–[28]. After nucleic acid extraction, 50 μl of eluate was concentrated with the SpeedVac vacuum concentrator (Eppendorf, Hamburg, Germany) and dissolved in 10 μl fragmentation master mix (NEBNext). The NEBNext Ultra II Directional RNA Library prep kit (New England Biolabs, Ipswich, MA, USA) for Illumina was used for RNA library preparation, incorporating several alterations to the manufacturer’s protocol to be able to detect both DNA and RNA in the sample. Specifically, poly-A mRNA capture isolation, rRNA depletion, and DNase treatment steps were omitted and dual indexed adaptors were used. The SeqCap EZ HyperCap probes (Roche, Basel, Switzerland) were designed in 2015 to cover 207 taxa genomes of viruses known to infect vertebrates including humans [29]. Recently, it has been shown that the probes cover > 99% of the SARS-CoV-2 genome [30] due to similarity with bat coronaviruses and the variability incorporated in the probe design. Viral DNA enrichment was performed using the SeqCap EZ HyperCap Workflow User’s Guide in pools of four amplified DNA libraries with overnight probe incubation. Washing and recovering captured DNA were performed using the HyperCap Target Enrichment kit and HyperCap Bead kit. Lastly, postcapture PCR amplification was performed with KAPA HiFi HotStart ReadyMix (2X) and Illumina NGS primers following manufacturers’ instructions, followed by AMPure bead purification. The quality and quantity of the postcapture multiplexed libraries were assessed by Fragment Analyzer (Agilent) or Bioanalyzer (Agilent, Santa Clara, CA, USA). Sequencing was performed on the NovaSeq 6000 system (Illumina, San Diego, CA, USA) obtaining approximately 10 million 150 bp paired-end reads per samples.

### Data analyses

In order to minimize potential differences resulting from variation in analysis tools and settings, a uniform pipeline consisting of steps for QC, trimming, mapping, and variant calling was used for sequence data from Illumina and Ion platforms (Supplementary Fig. 1). Illumina and Ion platform samples were processed in two different centers, every center using a marginally different mapping protocols. For ONT data, platform-specific mapping and variant calling tools handling higher error rates were used to gain optimal results from this type of dataset.

#### Illumina data from AmpliSeq, EasySeq, and viral metagenomic protocols

Demultiplexing was performed according to Illumina manufacturer protocol using bcl2fastq v2.20 (Illumina). Removal of duplicate reads was not performed since unique molecular identifiers (UMIs) in principle were not compatible with the nonrandom, tiled amplicon-based WGS protocols in the current study and were thus not incorporated in any of the wet lab procedures described here. Quality control and trimmings per read were performed utilizing Trimmomatic v0.36 (“LEADING:3 HEADCROP:31 TRAILING:3 SLIDINGWINDOW:4:15 MINLEN:40”) [31]. To remove and count the number of sequence read mapping to the human genome, reads were mapped to GRCh38 using Bowtie2 v2.1.0 (“–local–qc-filter–quiet”)[32]. Unmapped reads were subsequently mapped to the SARS-CoV-2 genome NC_045512.2 [33]. Mapped reads were indexed in a genome sorted bam file by SAMtools v1.7 [34] [35]. Variant calling was done using BCFtools v.1.7 (“bcftools call–ploidy 1-v-m”) [36].

#### Ion AmpliSeq data

Primer-removed fastq files were exported for further analysis using the Torrent Suite Software (Thermo Fisher Scientific, The Netherlands). Per read quality control was performed using Trimmomatic v0.36 (“LEADING:3 HEADCROP:31 TRAILING:3 SLIDINGWINDOW:4:15 MINLEN:40”) [31]. The resulting quality checked reads were first mapped to the human reference genome HG19 using BWA v0.7.17 [37] with default settings (“bwa bwasw”) to remove all reads of potential human origin. Unmapped reads were subsequently mapped to the SARS-CoV-2 reference genome Wuhan-Hu-1 [38]. The resulting sequence alignment map (SAM) files were converted to BAM, sorted, and indexed using SAMtools v1.14 [34][34]. Variant calling was performed using BCFtools v.1.7 (“bcftools call–ploidy 1-v-m”) [36].

#### ONT custom primer data

Demultiplexing was performed using Porechop v0.2.4 [34]. Primers were trimmed using Cutadapt v3.0 [39]. Reference-based alignment was carried out using Minimap2 v2.17-r941 [40] against both the human genome GRCH38 and SARS-CoV-2 genome NC_045512.2 [33]. Variant calling was performed by filtering of variants using the Python module Pysam v 0.16.0.1 [41].

### Performance and statistical analyses

Mapping coverage was analyzed using a threshold of 10 × depth per base for all platform data except for ONT data, where a 20 × depth per base was considered as threshold to ensure reliable variant calling as was previously described in literature [42]. Coverages per base were calculated using SAMtools v1.7 [34] [35] with the corresponding depth option. Correlation between genome coverage percentage and Ct values was calculated using Spearman’s rho [43]. Read mapping quality and base quality (phred) were computed using SAMtools v.11 [34] [35] with the coverage option. High mapping quality represents a more unique alignment, and low mapping quality represents a marginal difference between the alignment and the best secondary alignment option within the reference. High phred scores represent accurate base calling.

### Phylogenetic trees

Maximum likelihood trees of the consensus genomes from all methods were generated using the SAMtools consensus option (setting “mpileup-d 10” and for ONT “mpileup-d 20”) [29], Clustal Omega v1.2.4 (“clustal omega-t DNA”) [44], and FastTree v2.1.11 (“FastTreeMP-nt-gtr”) [45] [46]. Consensus genomes with ≥ 98% genome coverage were included, genome coverages based on minimal 10 × read depth for all methods, and 20 × read depth for ONT sequencing. Variant frequencies of > 50% were implemented in the consensus genome, though error profiles, like those of ONT, and short insertions/deletions (indels) not consistently called by SAMtools can lead to an inaccuracy of the consensus.

## Results

In total, 26 clinical samples from 24 patients were sequenced using the five SARS-CoV-2 sequencing protocols included in the current comparison (Fig. 1). Hands-on time, sequencing run time, and material costs per method are shown. Additional protocol characteristics are listed in Suppl. Table 1. The breadth of genome coverage, depth of genome coverage, proportion of SARS-CoV-2 reads, and performance of variant calling were compared.

### Quality performance

To assess the mapping quality scores, representing the probability that a read was misaligned, median mapping quality scores were analyzed (Suppl. Table 2). The mapping quality for all protocols was higher than 40, which equals a mapping accuracy of 99.99%. The median base quality (phred) scores reflecting the estimates of errors emitted by the sequencing platforms ranged from Q23.8 (ONT, *P*_error_ 0.004%) and Q26.6 (Ion, *P*_error_ 0.002%) to Q36 for Illumina protocols (*P*_error_ 0.0003%).

### Genome coverage

**Fig. 2:**
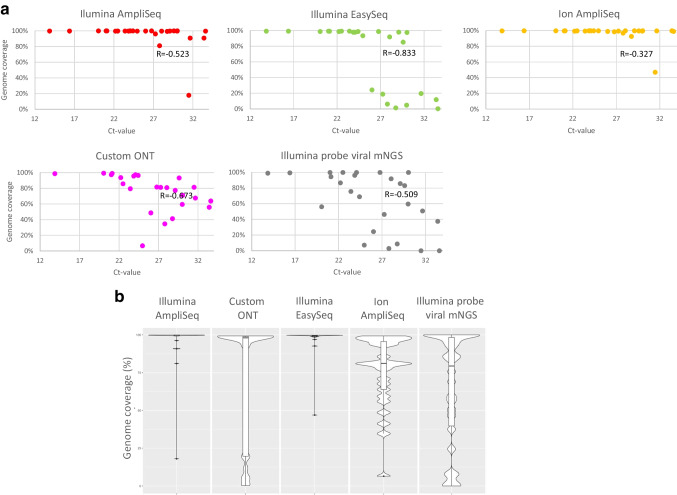
Proportion of SARS-CoV-2 genome coverage of sequencing reads using the five protocols compared. The scatter plots (**a**) indicate the SARS-CoV-2 genome (NC_045512.2) coverage per PCR Ct values, each dot represents a single sample. A threshold of 10 × depth per base was considered for all platform data except for ONT data, where a 20 × depth per base was considered as threshold ensuring reliable variant calling. $R$ values represent Spearman’s correlation coefficient (rho). The violin plots (**b**) indicate the distribution of the proportion covered per protocol, horizontal markers indicate the median, and the interquartile range

Median genome coverages (%) and median read depth of all protocols are listed in Table 1, with median values of samples with Ct values below 30 specified. SARS-CoV-2 genome coverages per protocol per sample are shown in Fig. 2, and more detailed information, including mean base and mean mapping quality scores, is presented in Suppl. Table 2. As anticipated, amplicon-based protocols generally resulted in higher genome coverage rates compared to the probe hybridization-based metagenomic protocol, though median genome coverages using the custom primer ONT protocol were within the same range for samples with Ct values of ≤ 30 (81.2% for ONT and 86.7% for mNGS, Suppl. Table 2). The median genome coverage across the other three amplicon-based protocols was comparable for samples with Ct values of ≤ 30, respectively, 99.7% and 99.8% when using the Ion AmpliSeq and the Illumina AmpliSeq protocol, followed by the EasySeq protocol for Illumina (98.05%, Table 1). An increase in Ct values resulted in only limited reduction of genome coverage when using the Ion AmpliSeq ($R = -0.327$) and Illumina AmpliSeq ($R = -0.523$) protocols. When considering all samples, including high Ct values, the genome coverage differed greatly between the amplicon-based protocols.

**Table 1:** Overview of median SARS-CoV-2 genome coverages (%) and read depth per protocol

	Illumina AmpliSeq		Illumina EasySeq		Ion AmpliSeq		Custom primers ONT		Illumina probe viral mNGS	
Median	99.80%	1.313	98.00%	316	99.65%	2.080	81.20%	860	75.70%	2.813
Median Ct ≤ 30	99.80%	3.339	98.05%	410	99.70%	2.205	81.60%	789	86.70%	6.277
Mean Ct ≤ 30	98.77%	4.521	77.71%	554	99.13%	3.257	77.05%	748	71.46%	17.553

The median read depth of coverage per position ranged from 316 when using the Illumina EasySeq protocol to 860 when using ONT and > 2000 for the Ion AmpliSeq and the probe hybridization-based metagenomic protocol. This depended on the throughput of the platform and kit, the total number of reads requested, and the number of samples multiplexed.

### SARS-CoV-2 amplicon balance

**Fig. 3:**
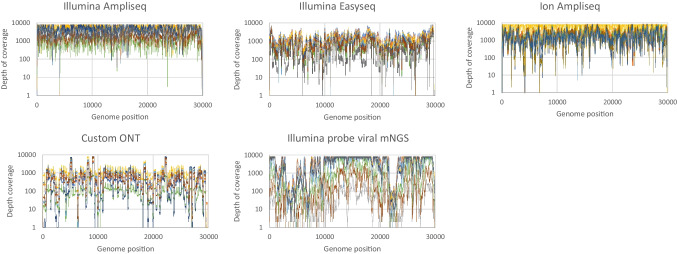
Distribution of sequence read depth over the SARS-CoV-2 genome using the five protocols compared. The number of sequence reads (logarithmic scale) per SARS-CoV-2 genome (NC_045512.2) position, using the five protocols compared. A selection of nine samples with higher viral loads (Ct values ranging from 13 to 23) is visualized. Each color represents an individual sample

The SARS-CoV-2 amplicon balance was assessed by evaluating the distribution of sequence reads across the SARS-CoV-2 genome. The average read depth per genome position was computed for a selection of nine samples with the highest viral loads (Ct values ranging from 13 to 23) (Fig. 3). When comparing the genome coverage profiles across the five protocols, distinct signatures were observed for each method. The read depth was most even when using the Illumina AmpliSeq protocol, in contrast to the uneven depth obtained using the probe hybridization-based protocol. The difference in depth between depth of coverage peaks and dips varied generally 2 log_10_-fold when using the Illumina AmpliSeq protocol, up to 4 log_10_-fold for the probe-based viral metagenomic protocol. When examining the differences in read depths in more detail, certain positions had protocol dependent, structural lower read depth for multiple samples. An example of a protocol with a structural drop of depth (to 0–11X read depth per sample) was observed at genome position 4117–4149 (ORF1a) when using the Illumina AmpliSeq and Ion AmpliSeq protocols. These findings were indicative of a primer failure caused by a specific SNV. The custom ONT protocol resulted in several samples with a low read depth in the amplicons spanning the regions 2690–2715 and 6260–6490 (ORF1a). Hybridization probe viral mNGS resulted in the largest regions with low coverage, especially regions 1000–10,000 (ORF1a) and 22,250–23,000 (Spike), with the last one at risk for missing mutations in the spike protein.

### Variant calling and phylogenetic analysis

**Fig. 4:**
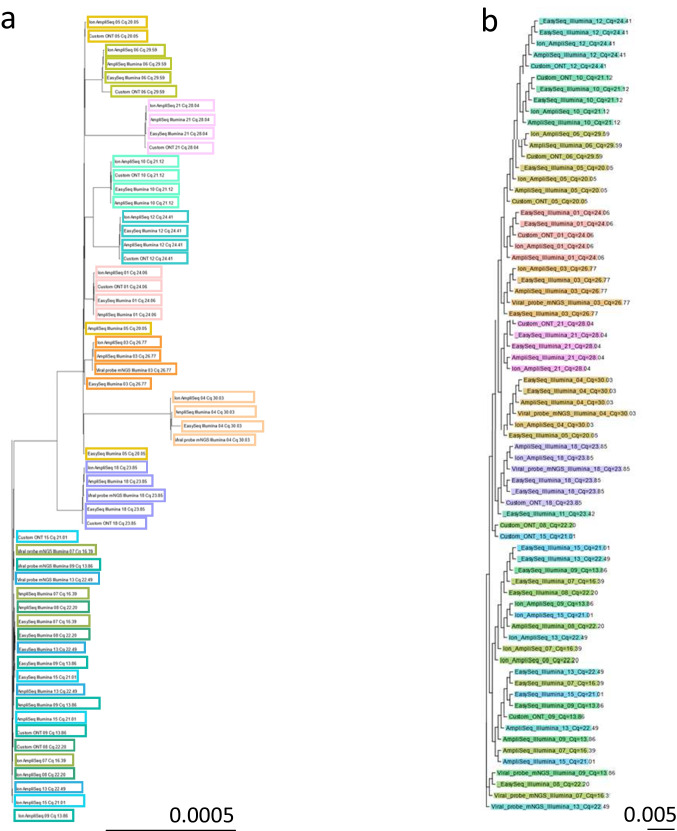
Tree of likelihood ratios based on consensus sequences of samples with genome coverages of ≥ 98% for each of the protocols. Phylogenetic trees were build based on consensus sequences resulting from each of the protocols (FastTree [45] [46] and IQtree [59]). For readability, a magnification is shown that includes samples with ≥ 98% genome coverage for four or more of the protocols (14 samples). A threshold of 10 × depth per base was considered for all platform data except for ONT data, where a 20 × depth per base was considered. Each color represents an individual sample. Clustering was independent of the protocol **a** IQTree, gtr [59]; **b** however, when gaps in the sequences (deletions and uncovered positions) were masked instead of considered as matches, in cases of closely related sequences (lower part of the tree), clustering per protocol was also detected

To assess the performance of variant calling across the protocols, consensus sequences were aligned to the SARS-CoV-2 reference NC_045512.2; SNVs detected per protocol are depicted in Suppl. Table 3. Consensus sequences were used to build a phylogenetic tree for samples in which ≥ 4 protocols had a genome coverage of 98% and higher ($n=14$ samples). In the phylogenetic tree where gaps in the sequence (uncovered positions and indels) were considered a match with the reference sequence (Fig. 4a), consensus genomes of specific samples clustered independent of the used protocol and analysis pipeline. However, when gaps were simply masked in the pairwise comparison (affecting solely the denominator, the total number of positions counted), for highly identical sequences (lower part of the tree), some per protocol clustering was also observed across Illumina, Ion, ONT, and probe-based technologies, up to 0.005 substitutions/site distances between methods (Fig. 4b). These findings indicate the effect of gaps in sequences in relation to the type of cluster analyses in case of highly identical sequences.

### SARS-CoV-2 sequencing efficiency: proportion of SARS-CoV-2 reads

**Fig. 5:**
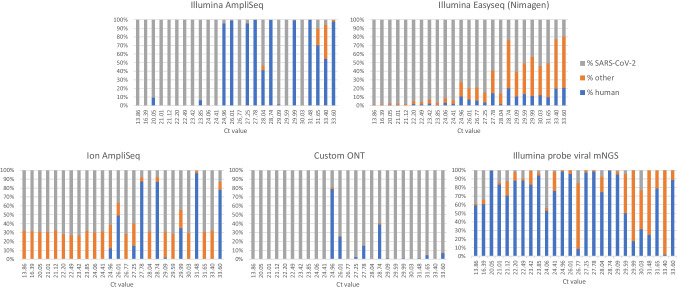
Proportion of SARS-CoV-2 read counts, compared to human and other (bacterial) read counts. The proportion of SARS-CoV-2, human, and other read counts is shown for each of the five protocols. Each bar (PCR-Ct value) represents an individual sample

To assess the efficiency of the protocols for sequencing SARS-CoV-2 genome in relation to background sequences, the proportion of SARS-CoV-2 read counts per sample, as opposed to human and other (bacterial) read counts, was computed (Fig. 5). As anticipated, the proportion of SARS-CoV-2 sequences was higher for amplicon-based protocols in comparison to the hybrid capture-based protocol, but differed considerably among the last. The proportion of SARS-CoV-2 specific reads varied from 73.72% on average when using the Illumina EasySeq protocol down to 8.19% on average when using the Illumina probe viral mNGS protocol. Mapping percentages of human reads ranged from 0.03% to 99.87% for Illumina and Ion Torrent amplicon-based protocols up to 69.98% on average for the Illumina probe viral mNGS protocol, with the long read ONT workflow resulting in the lowest number of human reads. Samples with an inefficient amplification, resulting in a low percentage of SARS-CoV-2 reads, showed a reverse pattern in the percentage of human reads (Fig. 5). Samples with a lower viral load demonstrated a greater number of human host reads while using an amplicon-based method. As can be deduced from these findings combined with Fig. 2, some protocols with lower SARS-CoV-2 sequence efficiency compensated for these results by deeper sequencing.

## Discussion

In this cross-platform benchmarking using clinical samples, the protocols differed with regard to the varying metrics studied. Each protocol had their own characteristics, advantages, and disadvantages. When considering genome coverage, the Illumina and Ion Torrent amplicon-based protocols were in favor and differences were detected even with the relatively small sample size. However, amplicon balance was not always even and showed protocol-specific drops. Protocols with uneven distribution of sequencing depth among amplicons may benefit from primer redesign or rebalancing of the primer pool to obtain a more even coverage threshold in difficult regions of the genome [42], and with emerging variants, primer designs need to be continuously monitored and updated to not have failing amplicons.

Phylogenetic analysis of SARS-CoV-2 was complicated as there were low numbers of differentiating mutations leading to a weak signal in inferring phylogeny [47]. Phylogenetic analysis indicated the effect of gaps in sequences in relation to the type of cluster analyses in case of highly identical sequences, possibly resulting from platform-associated effects such as deletion artefacts. This was in contrast to the setting of cluster analyses using sequences obtained using a single platform, since the likelihood of technology-associated characteristics in the sequences may be approximately evenly distributed over the samples. The SARS-CoV-2 sequence efficiency in relation to background sequences was the highest for the Illumina EasySeq protocol, comparable with the Ion AmpliSeq protocol, while the ONT protocol proportionally had the lowest number of human reads. Illumina EasySeq and the ONT protocol had the shortest hands-on time, with the latter additionally having the shortest sequence runtime and real-time data analysis.

As the pandemic continues worldwide and novel variants of interest and variants of concern continue to emerge [48] [49], genomic surveillance remains a critical component of the sustained management approach adhered to by the WHO [50]. Accordingly, the need for rapid SARS-CoV-2 genome sequencing protocols that can be easily adopted and automated and that are flexible and scalable remains crucial. Innovative protocol adaptations aiming at high-quality sequencing of low viral load samples (Ct values > 30) [11], inherent part of the diagnostic practice, have recently been reported, and such contributions may benefit the worldwide sequence community dedicated to surveillance. Implementation and compatibility of sequence regimes are influenced by characteristics of the local laboratory settings such as the availability of local resources and sequencing platforms with high or low-throughput nature. Reduction of the hands-on time needed for library preparation and overall turnaround time, scalability, and increased cost-efficiency of protocols would be beneficial in broader settings. Here, we aimed to provide data that can assist laboratories when selecting protocols for their local setting by comparing five platforms.

Drops in read depth of certain amplicons were detected in this study using different protocols. Regions with low read depth can result from (i) low amplicon coverage by design. High coverage regions have been correlated by coverage of multiple amplicons, whereas genome regions with coverage by only one amplicon resulted in low coverage [14]. Low read depth can also result from (ii) a SARS-CoV-2 variant resulting in primer mismatch in that particular amplicon, (iii) low efficiency of matching primers in multiplex reactions, or (iiii) an imbalance of the primer concentrations present in the multiplex. In our study, the length in bp of the drop in read depth assisted the distinction between single nucleotide variants resulting in a primer mismatch and low coverage by design as underlying cause. This illustrates the ongoing need of regularly updating primer kits due to arising mutants, as was done prior to testing for each of the WGS methods. Besides low coverage, another factor that can compromise SNV detection are primer-originated “contaminated” sequences that are PCR-amplified [14]. Wet lab methods and similarly bioinformatic tools can influence the performance of variant detection. Inaccurate trimming of primer sequences can mask or introduce SNVs located in the primer binding site; however, our study was not designed to detect such a phenomenon. Also, for example, Minimap2 [40], designed for analyses of sequences from relatively high error-rate platforms, allows considerable mismatches in the alignment with the reference sequence, whereas more stringent mapping tools can result in an absence of coverage in the mutated region. Differentiation of these type of effects resulting from analyses would require a design with cross-comparison of bioinformatic tools, which was not part of the current study though potential differences resulting from variation in analysis tools and settings were minimized using a uniform pipeline for sequence data from Illumina and Ion platforms. For ONT data, platform-specific mapping and variant calling tools handling higher error rates were used to gain optimal results from this type of dataset. In this comparison, no Pacific Biosciences protocol was tested, though this would have been a good addition, as the HiFi sequencing protocol generates accurate long reads that enables differentiation between viral sublineages [51].

Comparing the cDNA synthesis efficacy was not a subject of this study, as superscript III was used in the majority of the methods included. Reports comparing RTase efficiency indicate the most prominent differences in case of rare, challenging, and markedly human transcripts [52]. Given the low GC content of the SARS-CoV-2 genome (32–43%) and its unique low CG abundance [53] [54], it was anticipated that potential RTase efficiency differences would have only minor effects on our results, relative to the differences from other protocol steps. Finally, the current study was restricted by our sample collection time frame (2020), not containing later emerged mutants, and amount of available clinical material limiting testing more methods and retesting.

Viral (DNA/RNA) metagenomic sequencing has increasingly been adopted for pathogen diagnostics, microbiome analyses, and transcriptome analyses. Metagenomic methods work well for high-throughput sequencing of samples with high viral loads but here did not perform the most stable and accurate for low load SARS-CoV-2 samples. Importantly, SARS-CoV-2 sequencing was the original clinical request in 2020, at a time where commercial kits had not been developed yet. This exemplifies the benefit of the approach in earlier stages of pandemics. In later stages of the pandemic, it appeared beneficial to have protocols available which also work for lower viral load samples.

Importantly, with the above described pursuing emergence of variants, there is a vital need for sequencing-based approaches that tolerate mutations [55]. Probe capture-based approaches can tolerate large target sequence differences of ~ 10% or more from probe sequences [56] [57] in comparison with primer-based approaches. These characteristics have resulted in FDA emergency-use-authorization for hybridization-based SARS-CoV-2 genome sequencing in September 2021, in order to improve genomic surveillance of SARS-CoV-2 variants, for tracking viral evolution and guiding vaccine updates [58].

In summary, in this study, five cross-platform protocols for SARS-CoV-2 genome sequencing were benchmarked and evaluated on both technical performance and practicality. The results of our study build upon previous reports by providing additional comparison data testing Illumina, Ion Torrent, and ONT sequencing in parallel, incorporating technically innovative protocol steps including several analysis workflows. These data will be specifically of assistance for the sequence laboratories dedicated to ongoing surveillance efforts.

## Supplementary information

Below is the link to the electronic supplementary material.

Supplementary file1 (XLSX 15 KB)

Supplementary file2 (XLSX 21 KB)

Supplementary file3 (XLSX 29 KB)

Supplementary file4 (PPTX 356 KB)

## Data Availability

Raw data (excluding human reads) of the AmpliSeq SARS-CoV-2 sequencing (Illumina) and capture probe (Roche) with viral metagenomic NGS (Illumina) protocols have been uploaded to the NCBI Sequence Read Archive SRA (BioProject accession PRJNA943254) and are accessible via https://www.ncbi.nlm.nih.gov/bioproject/943254. Other datasets generated and analyzed during the current study are not publicly available due to consent restrictions but are available from the corresponding author on reasonable request.
