# Supplementary figures and images for: A comparison of five Illumina, Ion Torrent, and nanopore sequencing technology-based approaches for whole genome sequencing of SARS-CoV-2
Source: Eur J Clin Microbiol Infect Dis. 2023 Apr 5;42(6):701–13. doi: 10.1007/s10096-023-04590-0 (PMC10075175; doi:10.1007/s10096-023-04590-0)

## Slide 1
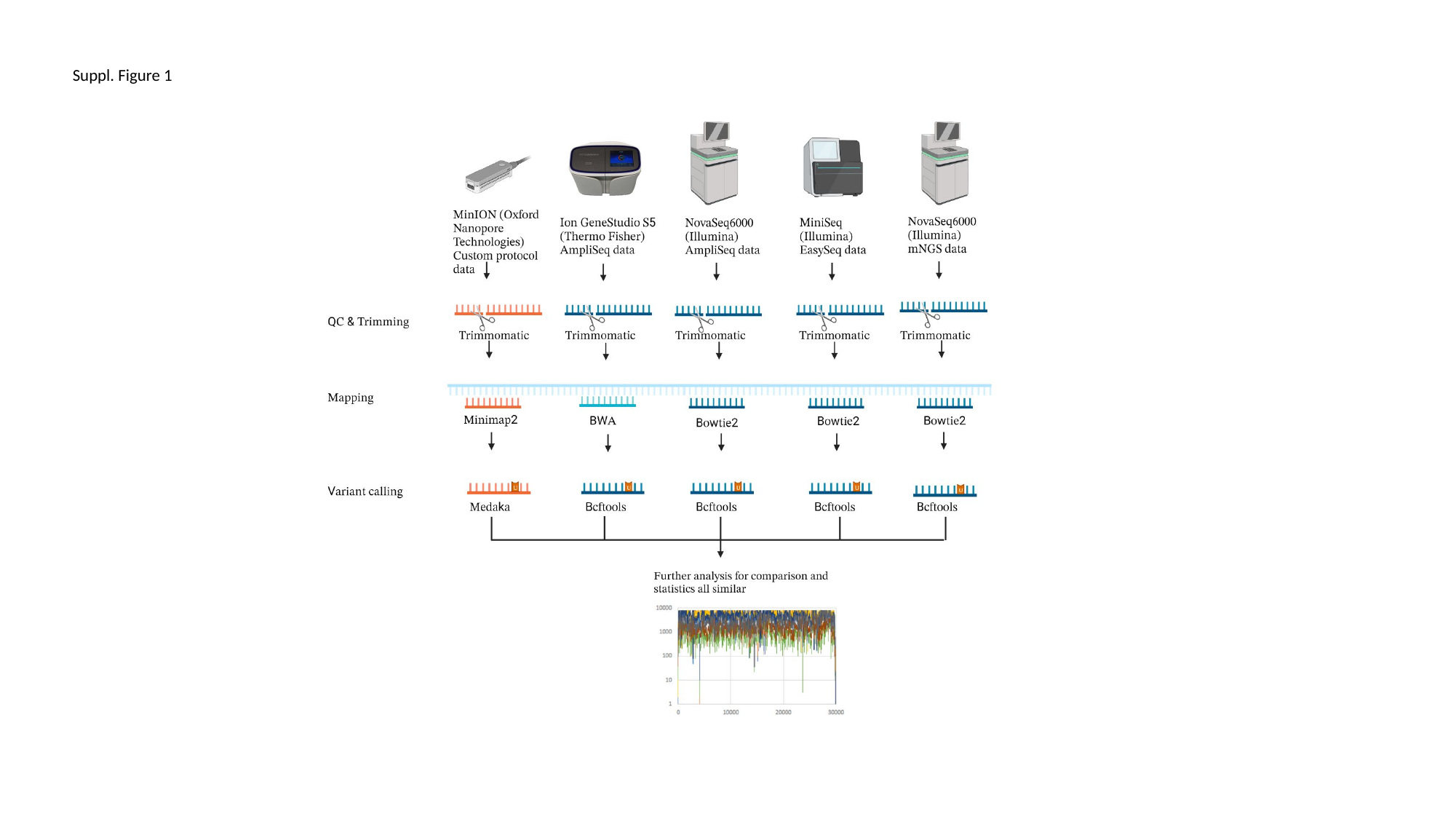

Suppl. Figure 1

Supplement: Supplementary file 4 — Supplementary file4 (PPTX 356 KB) [file 10096_2023_4590_MOESM4_ESM.pptx]
